# Importance of Both Fatness and Aerobic Fitness on Metabolic Syndrome Risk in Japanese Children

**DOI:** 10.1371/journal.pone.0127400

**Published:** 2015-05-20

**Authors:** Kensaku Sasayama, Eisuke Ochi, Minoru Adachi

**Affiliations:** 1 Joint Graduate School in Science of School Education, Hyogo University of Teacher Education, Kato, Hyogo prefecture, Japan; 2 Graduate School of Education, Okayama University, Okayama, Japan; Tulane School of Public Health and Tropical Medicine, UNITED STATES

## Abstract

Associations between body mass index (BMI), peak oxygen consumption (VO_2peak_), and metabolic syndrome (MetS) risk factors have not been adequately studied in Japanese children. Here the relationships between these parameters and the threshold aerobic fitness level necessary for low MetS risk were determined. The participants (299 children; 140 boys and 159 girls, aged 9.1 ± 0.3 years) were divided into four groups using the medians of predicted VO_2peak_ (_p_VO_2peak_) and BMI. MetS risk scores were calculated using z-scores. Receiver Operating Characteristic analysis was used to determine the threshold aerobic fitness level necessary for low MetS risk. The MetS risk score of the High BMI group was significantly higher than that of the Low BMI group for both sexes (p < 0.0001). However, the High BMI/High Fitness group had a significantly lower MetS risk score than the High BMI/Low Fitness group for both sexes. The _p_VO_2peak_ cut-off values for low MetS risk were 47.9 and 44.9 ml/kg/min for boys and girls, respectively. Our results suggest that improvements in both fatness and aerobic fitness are important for decreasing MetS risk. We also confirmed the _p_VO_2peak_ of cut-off values necessary for low MetS risk in Japanese children.

## Introduction

Metabolic syndrome (MetS) causes serious health issues in adults, including type 2 diabetes and cardiovascular disease [1–3]. According to the Third National Health and Nutrition Examination (NHANES III) and the NHANES 1999–2006 survey, the age-adjusted prevalence of MetS increased from 29.2% (1988–1994) to 34.2% (1999–2006) in U.S. adults [4]. In contrast, the prevalence of MetS among Japanese males and females from 2005 survey had been reported to be 19% and 7%, respectively [5].

The components of MetS, such as obesity, hyperglycemia, dyslipidemia, hypertension, and insulin resistance, in children and adolescents are similar to those in adults [6]. Although MetS occurs less often in children than in adults, several previous studies have established the prevalence of MetS (approximately 3.0%–4.0%) among children and adolescents [7–9]. These phenomena have also been reported in Japanese children [10–12].

MetS is associated with aerobic fitness and/or obesity in children [13,14]. The prevalence of MetS in overweight Hispanic children, for example, has been reported to be 30% [13]. The prevalence of MetS among overweight and obese Japanese children has been shown to be 8.7% and 17.7%, respectively [14]. Moreover, several studies have shown that higher aerobic fitness decreases MetS risk [15–19].

However, very few studies have demonstrated that MetS risk is associated with both fatness and aerobic fitness [20–23]. These studies have reported differences in MetS risk among four fatness and aerobic fitness groups in American, Australian, and Canadian children and adolescents [20–23]. Eisenmann et al. [20] showed that MetS risk was significantly lower in the high fitness and high fatness than in the low fitness and high fatness group in both boys and girls. However, a similar tendency was not consistently observed in other studies [21–23]. Thus, the MetS risk of children displaying high aerobic fitness with high fatness remains unclear.

Furthermore, the aerobic fitness level necessary for a low MetS risk needs to be studied. The aerobic fitness levels necessary for low MetS risk have been published for American, European, and Canadian children [16,24,25]. These studies determined the aerobic fitness threshold from aerobic fitness and MetS risk data using a receiver operating characteristic (ROC) analysis. Ruiz et al. [16] determined the aerobic fitness threshold (42.1 mL/kg/min and 37.0 mL/kg/min for 9–10 years old boys and girls, respectively) associated with low MetS risk using European (Estonia and Sweden) data. Adegboye et al. [24] (43.6 mL/kg/min and 37.4 mL/kg/min for 8–11 years old boys and girls, respectively) and Boddy et al. [25] (46.6 mL/kg/min and 41.9 mL/kg/min for 9–10.9 years old boys and girls, respectively) also determined the aerobic fitness thresholds associated with low MetS risk using European (Denmark, Estonia, Portugal, Norway, and U.K.) data. Although the 20-m shuttle run test level was relatively higher among Japanese children than among the children from 37 countries worldwide [26], the aerobic fitness level necessary for low MetS risk has not been determined for Japanese children.

The purposes of this study were to determine: 1) the differences in MetS risk among Japanese children categorized into four groups by fatness and aerobic fitness; and 2) the aerobic fitness level necessary to achieve a low MetS risk. We hypothesized that: 1) the risk of MetS is highest in Japanese children with low _p_VO_2peak_ and high BMI; and 2) The _p_VO_2peak_ level necessary for Japanese children to achieve a low MetS risk is higher than those observed in previous studies.

## Materials and Methods

### Participants

This cross-sectional, school-based study included 299 elementary school children. This group included 140 boys and 159 girls from 13 elementary schools in Japan. Data were collected from on 2008 in Ibara city, Okayama prefecture, Japan. The procedure conformed to the principles of the Declaration of Helsinki and was approved by the Institutional Review Board of the Okayama University. All participating children and their parents provided a written informed consent before participation.

### Instruments and Procedure

#### Anthropometric characteristics

Height and weight were measured in light clothing without shoes. Body mass index (BMI) was calculated using the ratio of weight (kg) to height (m^2^). Waist circumference (WC) was measured with a metal anthropometric tape midway between the lower rib margin and iliac crest. The WC to height ratio was calculated and reported as waist/height (W/H).

#### Aerobic fitness

Aerobic fitness was defined as the predicted peak oxygen consumption (_p_VO_2peak_) from a 20-m shuttle run test [27]. _p_VO_2peak_ was calculated using the method reported in a previous study [28].

#### Blood pressure

Systolic blood pressure (SBP) and diastolic blood pressure (DBP) were measured from the right arm using an automatic oscillometric method. SBP and DBP were taken from children in a seated, relaxed position after at least 10 min rest. Measurements were conducted between 9:30 and 12:00 h.

#### Blood samples

Plasma triglycerides (TG) and high-density lipoprotein cholesterol (HDL-c) levels were measured in a nonfasted state, between 9:30 and 12:00, when children were in elementary school using the Wako L-Type method (Wako Chemical, Japan).

### Data Analysis

Participant characteristics and MetS scores are reported as the mean ± S.D. MetS risk scores were calculated from the sums of sex-standardized values (z-scores) of the following six parameters: WC, W/H, TG, HDL-c, SBP, and DBP. A lower score indicates a lower MetS risk. Participants were divided into four groups using a median split of _p_VO_2peak_ and BMI; Low BMI/High Fitness (LB/HF), Low BMI/Low Fitness (LB/LH), High BMI/High Fitness (HB/HF), and High BMI/Low Fitness (HB/LF) groups. The association between the obtained variables was analyzed using the Pearson's correlation coefficient. One-way analysis of variance (ANOVA) was used to compare the MetS risk factors between the LB/HF, LB/LH, HB/HF, and HB/LF groups. Post-hoc analysis was performed using the Games–Howell test. Receiver Operating Characteristic (ROC) analysis was used to identify the threshold of aerobic fitness necessary to achieve a low MetS risk. A risk score in the bottom 75^th^ percentile was considered to indicate a low MetS risk. All analyses were performed using the SPSS Statistics software, version 20.0 (IBM, Armonk, NY). The level of significance was set to α = 0.05.

## Results

### Participants Characteristics

The descriptive characteristics of the study participants were 140 boys and 159 girls. Descriptive characteristics among two groups, or four groups, are shown in Tables 1 and 2, respectively (S1 Dataset).

**Table 1:** Participants characteristics and MetS risk factors by BMI categories.

		Boys		Girls	
		Low BMI	High BMI	Low BMI	High BMI
		n = 70	n = 70	n = 80	n = 79
Age	(years)	9.2	9.1	9.1	9.1
		(0.4)	(0.3)	(0.3)	(0.3)
Height	(cm)	133.2	138.0	135.2	136.9
		(4.9)	(5.9)	(5.4)	(5.4)
Weight	(kg)	27.0	37.5	27.6	34.5
		(2.7)	(8.6)	(2.8)	(5.2)
BMI	(kg/m^2^)	15.2	19.6	15.1	18.4
		(0.9)	(3.4)	(0.9)	(2.0)
${}_{\text{p}}{\dot{V}\text{O}}_{2\text{peak}}$	(ml/kg/min)	53.6	48.5	48.3	44.4
		(3.6)	(5.6)	(2.8)	(3.5)
WC	(cm)	54.5*	65.6	54.3*	62.0
		(3.1)	(9.6)	(3.3)	(6.4)
Waist / height		0.41*	0.47	0.40*	0.45
		(0.03)	(0.06)	(0.02)	(0.04)
TG	(mg/dl)	67.0*	84.7	76.1	88.7
		(37.2)	(47.5)	(42.2)	(59.0)
HDL-c	(mg/dl)	74.7*	66.5	69.9*	62.6
		(14.4)	(14.6)	(14.0)	(13.1)
SBP	(mmHg)	106.2*	110.5	108.4	109.6
		(8.5)	(8.3)	(9.6)	(9.8)
DBP	(mmHg)	60.0	60.8	61.8	61.4
		(9.0)	(7.6)	(8.1)	(8.5)

Values are means (S.D.)

Participants were divided into two groups using a median split of BMI; Low BMI and High BMI groups in each gender.

BMI; Body Mass Index, MetS risk score; metabolic syndrome risk score, WC; waist circumference

Waist / height; Waist to height ratio, TG; Triglycerides, HDL-c; high density lipoprotein cholesterol

SBP; systolic blood pressure, DBP; diastolic blood pressure

*; p<0.05 for differences between Low BMI and High BMI.

**Table 2:** Participants characteristics and MetS risk factors among BMI and _p_VO_2peak_ groups.

		Boys							Girls						
		Low BMI				High BMI			Low BMI				High BMI		
		High Fit		Low Fit		High Fit		Low Fit	High Fit		Low Fit		High Fit		Low Fit
		n = 47		n = 23		n = 23		n = 47	n = 58		n = 22		n = 21		n = 58
Age	(years)	9.2		9.1		9.1		9.1	9.1		9.2		9.1		9.1
		(0.4)		(0.3)		(0.3)		(0.2)	(0.3)		(0.4)		(0.4)		(0.3)
Height	(cm)	133.9		131.9		135.5		139.2	135.6		133.9		138.8		136.2
		(5.1)		(4.4)		(6.2)		(5.3)	(5.4)		(5.3)		(4.8)		(5.4)
Weight	(kg)	27.4		26.4		32.1		40.2	27.5		28.1		32.9		35.1
		(2.9)		(2.0)		(4.2)		(8.9)	(3.0)		(2.4)		(3.1)		(5.7)
BMI	(kg/m^2^)	15.2		15.2		17.4		20.6	14.9		15.6		17.1		18.8
		(0.9)		(0.8)		(0.9)		(3.7)	(0.9)		(0.4)		(0.8)		(2.1)
${}_{\text{p}}{\dot{V}\text{O}}_{2\text{peak}}$	(ml/kg/min)	55.6		49.6		54.1		45.7	49.5		45.3		48.8		42.9
		(2.6)		(1.4)		(1.8)		(4.7)	(2.4)		(0.8)		(1.7)		(2.5)
WC	(cm)	54.4	†,‡	54.9	§,||	59.2	¶	68.7	53.9	†,‡	55.6	§,||	58.6	¶	63.2
		(2.8)		(3.5)		(3.8)		(10.0)	(3.4)		(2.6)		(3.5)		(6.7)
Waist / height		0.41	†,‡	0.42	§,||	0.44	¶	0.49	0.40	*,†,‡	0.42	||	0.42	¶	0.46
		(0.02)		(0.03)		(0.02)		(0.07)	(0.03)		(0.02)		(0.02)		(0.04)
TG	(mg/dl)	67.7		65.6		73.8		90.0	76.6		74.8		62.6	¶	98.1
		(37.8)		(36.6)		(30.2)		(53.4)	(41.9)		(43.9)		(21.3)		(65.3)
HDL-c	(mg/dl)	75.7	‡	72.6		70.1		64.7	70.4	‡	68.6	||	69.9	¶	60.0
		(14.1)		(15.2)		(15.2)		(14.1)	(14.6)		(12.3)		(13.5)		(12.0)
SBP	(mmHg)	107.0	‡	104.6	||	107.0		112.1	107.3		111.5		110.2		109.3
		(7.9)		(9.4)		(8.2)		(7.9)	(9.4)		(9.4)		(9.4)		(10.0)
DBP	(mmHg)	59.9		60.2		59.7		61.3	61.2		63.2		58.9		62.3
		(10.0)		(6.8)		(8.1)		(7.3)	(7.3)		(10.0)		(8.1)		(8.5)

Values are means (S.D.)

Participants were divided into four groups using a median split of _p_VO_2peak_ and BMI; Low BMI/High Fitness (LH), Low BMI/Low Fitness (LL), High BMI/High Fitness (HH), and High BMI/Low Fitness (HL) groups.

BMI; Body Mass Index, MetS risk score; metabolic syndrome risk score, WC; waist circumference

Waist / height; Waist to height ratio, TG; Triglycerides, HDL-c; high density lipoprotein cholesterol

SBP; systolic blood pressure, DBP; diastolic blood pressure

*; p<0.05 Low BMI/High fit significantly different from Low BMI/Low fit

†; p<0.05 Low BMI/High fit significantly different from High BMI/High fit

‡; p<0.05 Low BMI/High fit significantly different from High BMI/Low fit

§; p<0.05 Low BMI/Low fit significantly different from High BMI/High fit

||; p<0.05 Low BMI/Low fit significantly different from High BMI/Low fit

¶; p<0.05 High BMI/High fit significantly different from High BMI/Low fit.

### Associations between aerobic fitness/BMI and each risk factor

Pearson's correlation analysis revealed that BMI was associated with WC (r = 0.933, P < 0.0001), W/H (r = 0.909, p < 0.0001), TG (r = 0.383, p < 0.0001), HDL-c (r = −0.430, p < 0.0001), and SBP (r = 0.372, p < 0.0001) in boys. In girls, BMI was also associated with WC (r = 0.871, p < 0.0001), W/H (r = 0.860, p < 0.0001), TG (r = 0.277, p < 0.0001), HDL-c (r = −0.266, p = 0.0007), and SBP (r = 0.165, p = 0.0381). Also, we confirmed similar results in the High BMI group but the Low BMI group was only associated with WC and W/H in boys and with WC, W/H and SBP in girls. Aerobic fitness in boys was associated with WC (r = −0.784, p < 0.0001), W/H (r = −0.771, p < 0.0001), TG (r = −0.318, p < 0.0001), SBP (r = −0.288, p = 0.0006), and HDL-c (r = 0.421, p < 0.0001). Aerobic fitness in girls was also associated with WC (r = −0.652, p < 0.0001), W/H (r = −0.709, p < 0.0001), TG (r = −0.377, p < 0.0001), and HDL-c (r = 0.322, P < 0.0001). However, both aerobic fitness and BMI were not associated with DBP in boys or girls. In addition, aerobic fitness was not associated with SBP in girls.

### MetS risk factors and scores by BMI category

**Fig 1:**
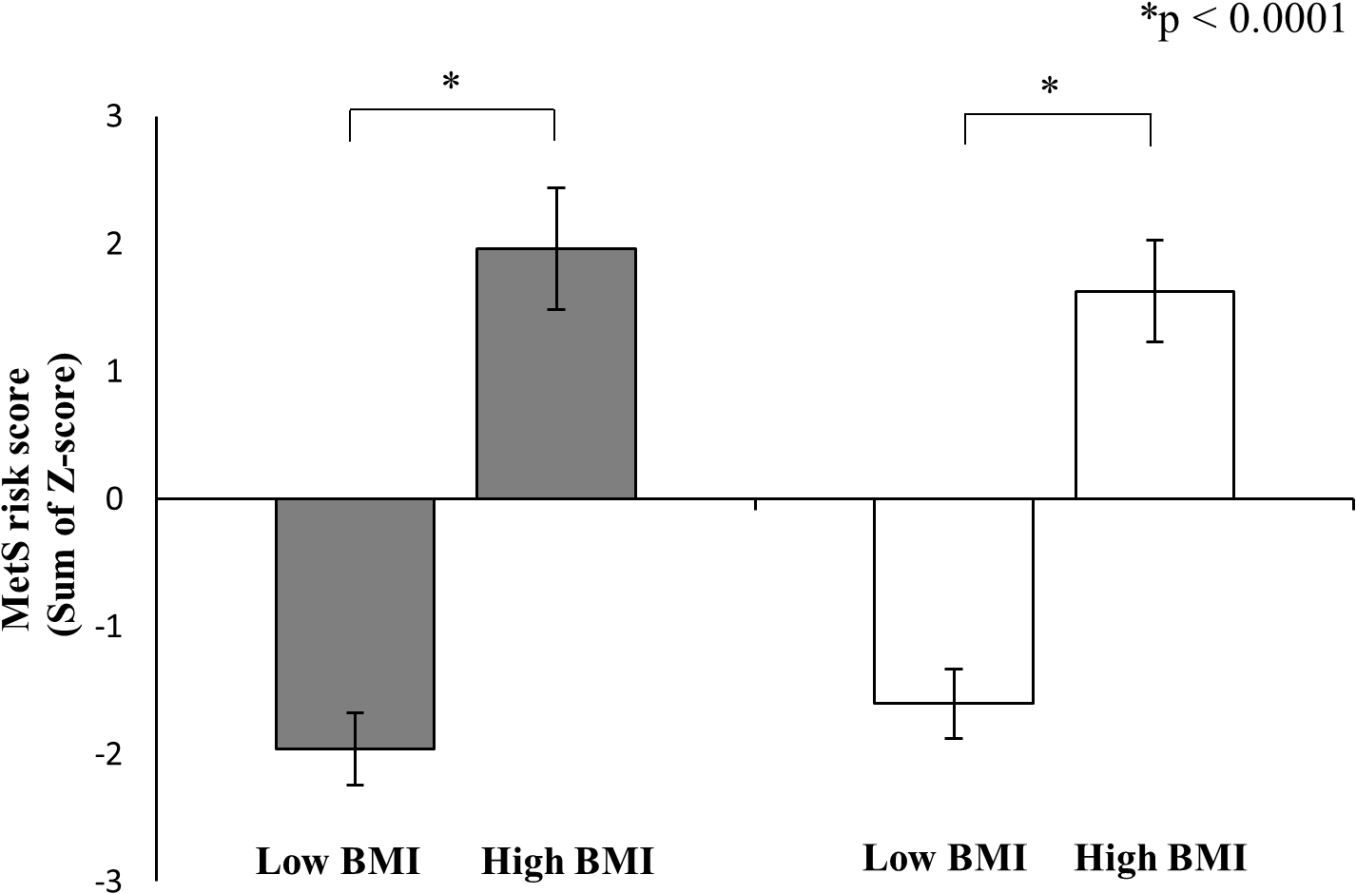
MetS risk score by BMI categories in boys (black bar) and girls (white bar). Values are means S.E. MetS risk scores were calculated from the sums of sex-standardized values (z-scores) of the following six parameters: WC, W/H, TG, HDL-c, SBP, and DBP. For significance notations see the figure. The results showed that high BMI/low Fit group had the highest MetS risk score in both sexes (boys: p<0.05, girls: p<0.05). MetS risk score was significantly higher in the High BMI group than Low BMI group in both sexes (p<0.0001). Abbreviations: MetS risk score, metabolic syndrome risk score; BMI, body mass index.

Results comparing MetS risk factors by BMI category are shown in Table 1. WC and W/H were significantly higher in the High BMI group than in the Low BMI group and HDL-c was significantly lower in the High BMI group than in the Low BMI group for boys and girls. TG and SBP were significantly higher in the High BMI group than in the Low BMI group for boys. Fig 1 shows the MetS risk scores by BMI category. The MetS risk score of the High BMI group was significantly higher than that of the Low BMI group for both genders (p < 0.0001).

### MetS risk factors and MetS risk score across BMI/_p_VO_2peak_ groups

**Fig 2:**
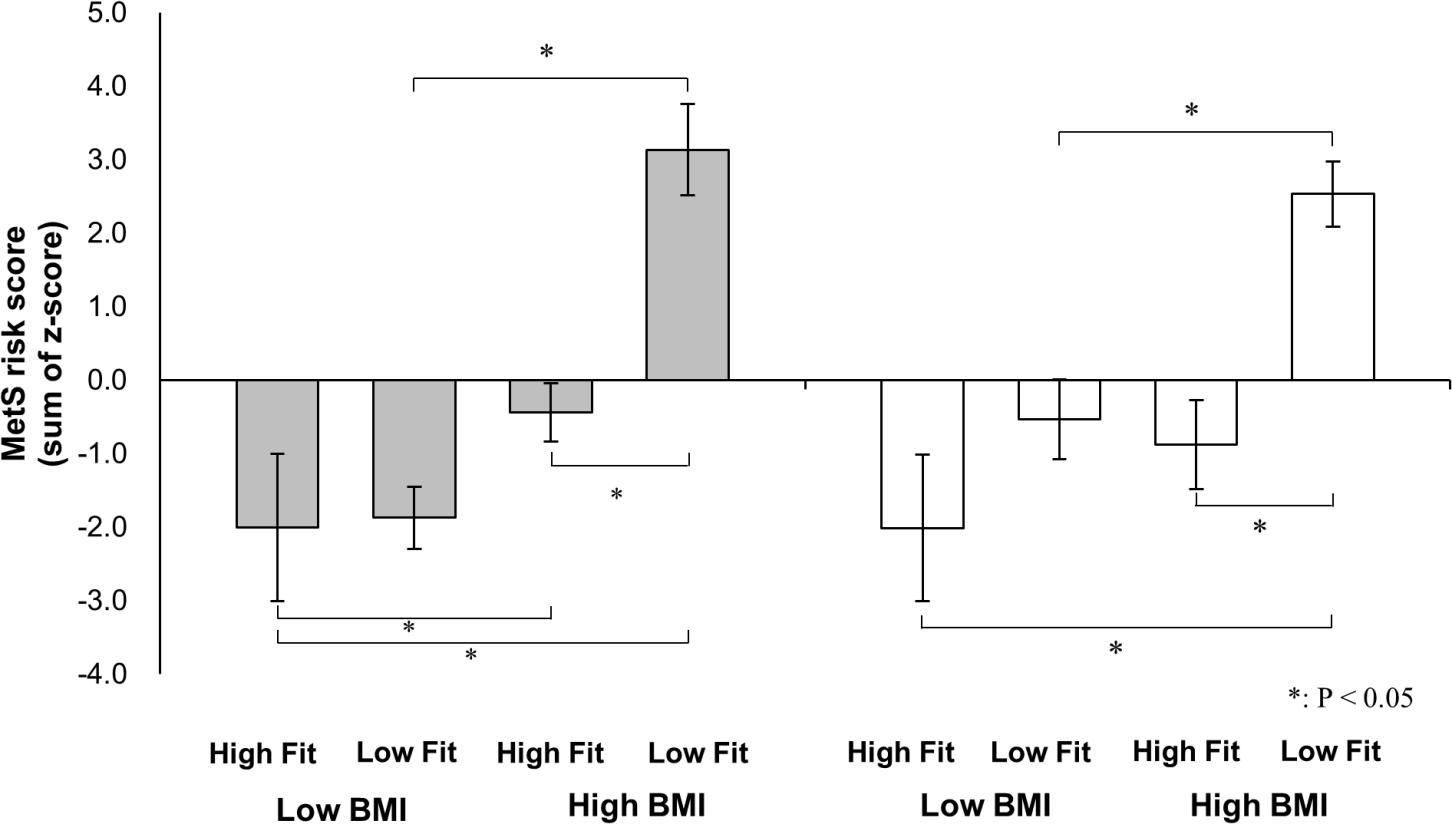
Differences in the metabolic syndrome risk score across BMI and _p_VO_2peak_ groups in boys (black bar) and girls (white bar). Values are means S.E. MetS risk scores were calculated from the sums of sex-standardized values (z-scores) of the following six parameters: WC, W/H, TG, HDL-c, SBP, and DBP. For significance notations see the figure. The results showed that high BMI/low Fit group had the highest MetS risk score in both sexes (boys: p<0.05, girls: p<0.05). Abbreviations: MetS risk score, metabolic syndrome risk score; BMI, body mass index.

Results comparing MetS risk factors across the four BMI/_p_VO_2peak_ groups are shown in Table 2. Significant differences in WC, W/H, HDL-c, and SBP were observed among these four groups in boys. In boys, the WC in the HB/LF group was significantly higher than that in the other three groups (p < 0.0001). W/H in the HB/LF group was also significantly higher than that in the other three groups (p < 0.0001). The HDL-c level in the HB/LF group was significantly lower than that in the LB/HF group. Finally, the SBP of the HB/LF group was significantly higher than that of the LB/HF and LB/LF groups. We also confirmed significant differences in WC, W/H, TG, and HDL-c in girls. The TG level in the HB/HF group was significantly lower than that in the HB/LF group. Fig 2 shows the results of the MetS risk score calculations. The HB/LF group had the highest MetS risk score in both boys (p < 0.0001) and girls (vs. LB/HF, p < 0.0001; vs. LB/LF, p = 0.0003; vs. HB/HF, p = 0.0002). In addition, the MetS risk score of the HB/HF group was significantly lower than that of the HB/LF group in both boys and girls.

### ROC analysis

**Fig 3:**
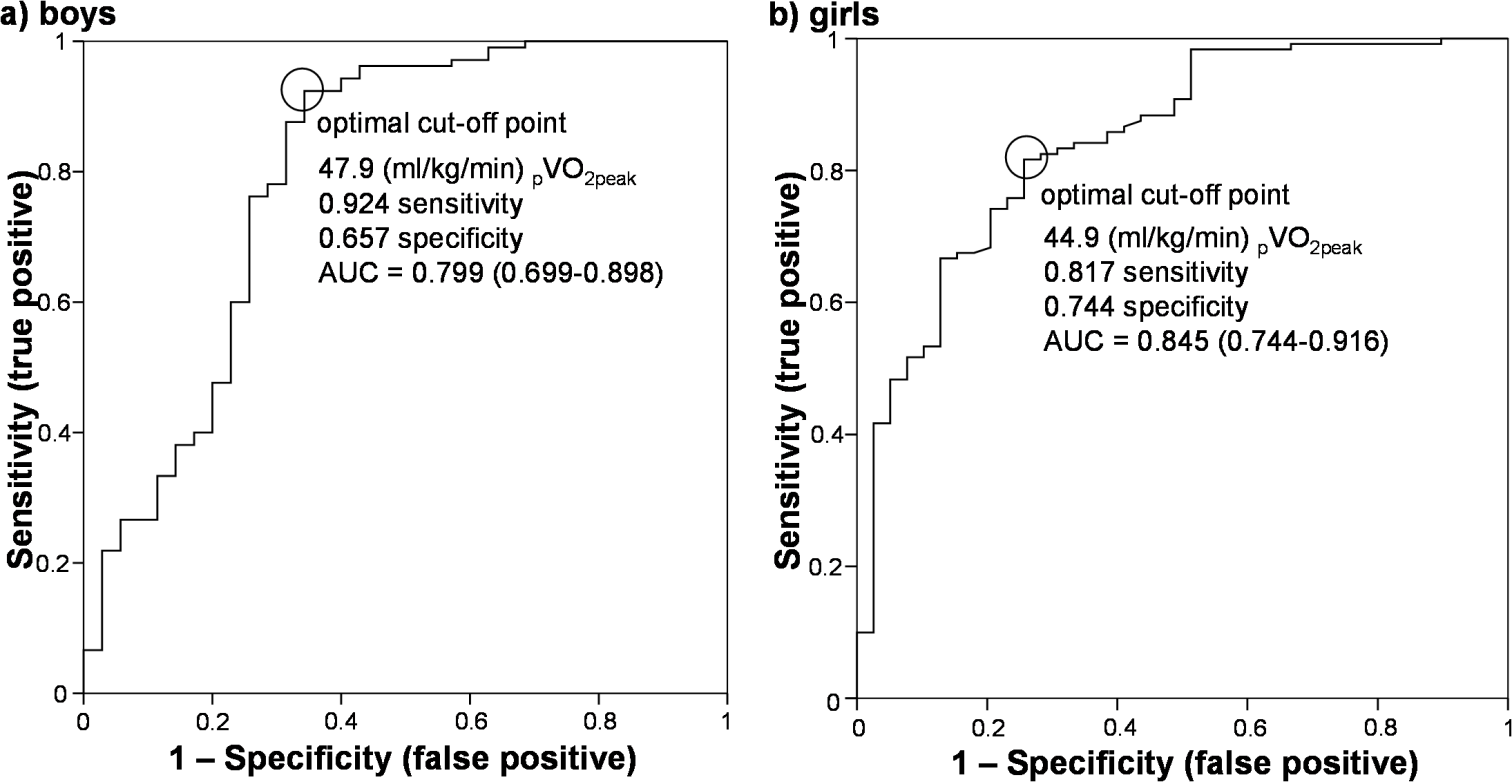
Receiver operating characteristic (ROC) analysis to identify low/high metabolic risk score in boys and girls. ROC curves showing the optimal _p_VO_2peak_ cut-off point for metabolic syndrome risk score of <75%ile in boys and girls. AUC indicates the area under the curve (95% confidence interval). In boys, the optimal pair of true- and false-positive rates were 0.92 and 0.66, respectively (AUC = 0.80%; 95% confidence interval (CI): 0.70–0.90; p < 0.0001). In girls, these parameters were 0.82 and 0.74, respectively (AUC = 0.85%; 95% CI: 0.74–0.92; p < 0.0001). _p_VO_2peak_ of cut-off value for low metabolic risk was 47.9 and 44.9 mL/kg/min in boys and girls, respectively.

Fig 3 shows the results of the ROC analysis. The ROC analysis demonstrated the significant discriminating ability of _p_VO_2peak_ for identifying low versus high MetS risk scores in boys (AUC = 0.80%; 95% confidence interval CI: 0.70–0.90; p < 0.0001), and girls (AUC = 0.85%; 95% CI: 0.74–0.92; p < 0.0001). _p_VO_2peak_ at these points were 47.9 and 44.9 ml/kg/min in boys and girls, respectively.

## Discussion

The purposes of this study were to investigate the relationships between BMI, aerobic fitness, and MetS risk factors and determine the threshold aerobic fitness level necessary to achieve a low MetS risk in Japanese children. The results demonstrate that the High BMI/Low fitness group had the highest MetS risk score in both boys and girls. The MetS risk score of the High BMI/High fitness group was significantly lower than that of the High BMI/Low fitness group in both genders. The _p_VO_2peak_ cut-off values for low metabolic risk was 47.9 and 44.9 mL/kg/min for boys and girls, respectively.

In addition, the results demonstrate that children in the Low BMI group had lower MetS risk factors and MetS risk scores, on average, than those in children in the High BMI group from Pearson's correlation analysis and ANOVA. This finding is consistent with those of previous studies assessing the relationship between BMI/%Fat and MetS risk in European, American, Australian and Canadian children and adolescents [20–23,29]. We confirmed that an increase in BMI was associated with higher MetS risk.

Furthermore, MetS risk is also associated with aerobic fitness. In adults, high levels of aerobic fitness among those with high BMI decrease cardiovascular disease risk [30,31]. However, these phenomena have not been consistently observed in children and adolescents [20–23]. Our results clearly show that, among those with high fatness, the children in the high aerobic fitness group have lower MetS risk scores. This finding was also observed for WC and W/H in boys and WC, W/H, TG, and HDL-c in girls. We suggest that a high level of aerobic fitness is important for decreasing MetS risk, even among overweight and obese children. With regard to the reason of lower MetS risk score in High BMI/High fit vs. High BMI/Low fit, it may be related to physical activity [17,32,33]. In addition, there is a possibility that body composition may have a role in MetS risk. More importantly, MetS risk score in boys was lower in High BMI/High fit than Low BMI/High fit but that in girls was not. Therefore, in children with high fitness, it is suggested that a gender difference could exist. Based on the present observations, we speculate that in high fitness groups, BMI may have a stronger relationship with MetS risk in boys compared to girls. In the future, it is necessary to investigate gender difference in MetS risk in concomitant with physical activity and/or body composition.

The aerobic fitness levels found to be necessary for a low MetS risk in this study (47.9 and 44.9 ml/kg/min for 9–10 years old in boys and girls, respectively) were relatively higher than those reported in previous studies by Ruiz et al. [16] (42.1 and 37.0 ml/kg/min for 9–10 years old boys and girls, respectively), Adegboye et al. [24] (43.6 and 37.4 ml/kg/min for 8–11 years old boys and girls, respectively) and Boddy et al. [25] (46.6 and 41.9 ml/kg/min for 9–10.9 years old boys and girls, respectively). In the studies of Ruiz et al. [16] and Adegboye et al. [24], aerobic fitness was evaluated using a cycle-ergometer test. On the other hand, in our study and that of Boddy et al. [25], aerobic fitness was evaluated using the 20-m shuttle run test. These differences in aerobic fitness threshold may reflect the method used to evaluate aerobic fitness. However, in Japanese children, the 20-m shuttle run test level was higher than that observed among 37 countries worldwide [26]. Therefore, it is reasonable to expect that our results would be higher than those observed in previous studies.

This study has several limitations. First, in terms of safety and ethical considerations, blood samples were measured in a nonfasting state. Therefore, we were not able to measure glucose or insulin. However, we believe the data are sufficient to provide the first determination of a _p_VO_2peak_ cut-off value for low MetS risk in Japanese children. These data are quite important for the children. Second, this study was that VO_2peak_ was predicted from a single 20-m shuttle run. Standard exercise testing methods with directly measured oxygen consumption are required to confirm our findings. Third, this study was performed in only one city in Japan. However, our study covered all 13 schools within that city. The VO_2peak_ of cut-off value we found necessary for low MetS risk in Japanese children needs to be confirmed using a large sample size. Finally, because this study is cross-sectional, a longitudinal analysis is required to better understand the importance of fatness and aerobic fitness on MetS risk.

## Conclusions

This study suggests that improvements in both BMI and aerobic fitness are important for achieving a low MetS risk. Moreover, we confirm specific _p_VO_2peak_ of cut-off values (47.9 and 44.9 ml/kg/min in boys and girls, respectively) for low MetS risk in Japanese children.

## Supporting Information

S1 Dataset Dataset of all parameters from140 boys and 159 girls. (XLSX)

## References

[1] Eckel RH, Grundy SM, Zimmet PZ. The metabolic syndrome. Lancet. 2005;365: 1415–1128. 15836891 10.1016/S0140-6736(05)66378-7

[2] Hanson RL, Imperatore G, Bennett PH, Knowler WC. Components of the “metabolic syndrome” and incidence of type 2 diabetes. Diabetes. 2002;51: 3120–3127. 12351457 10.2337/diabetes.51.10.3120

[3] Lakka HM, Laaksonen DE, Lakka TA, Niskanen LK, Kumpusalo E, Tuomilehto J, et al The metabolic syndrome and total and cardiovascular disease mortality in middle-aged men. JAMA. 2002;288: 2709–2716. 12460094 10.1001/jama.288.21.2709

[4] Mozumdar A, Liguori G. Persistent increase of prevalence of metabolic syndrome among U.S. adults: NHANES III to NHANES 1999–2006. Diabetes Care. 2011;34: 216–219. 10.2337/dc10-0879 20889854 PMC3005489

[5] Ishizaka N, Ishizaka Y, Toda E, Hashimoto H, Nagai R, Yamakado M. Hypertension is the most common component of metabolic syndrome and the greatest contributor to carotid arteriosclerosis in apparently healthy Japanese individuals. Hypertens Res. 2005;28: 27–34. 15969252 10.1291/hypres.28.27

[6] Cruz ML, Goran MI. The metabolic syndrome in children and adolescents. Curr Diab Rep. 2004;4: 53–62. 14764281 10.1007/s11892-004-0012-x

[7] Raitakari OT, Porkka KV, Rönnemaa T, Knip M, Uhari M, Akerblom HK, et al The role of insulin in clustering of serum lipids and blood pressure in children and adolescents. The Cardiovascular Risk in Young Finns Study. Diabetologia. 1995;38: 1042–1050. 8591817 10.1007/BF00402173

[8] Chen W, Srinivasan SR, Elkasabany A, Berenson GS. Cardiovascular risk factors clustering features of insulin resistance syndrome (Syndrome X) in a biracial (Black-White) population of children, adolescents, and young adults: the Bogalusa Heart Study. Am J Epidemiol. 1999;150: 667–674. 10512420 10.1093/oxfordjournals.aje.a010069

[9] Cook S, Weitzman M, Auinger P, Nguyen M, Dietz WH. Prevalence of a metabolic syndrome phenotype in adolescents: findings from the third National Health and Nutrition Examination Survey, 1988–1994. Arch Pediatr Adolesc Med. 2003;157: 821–827. 12912790 10.1001/archpedi.157.8.821

[10] Okada T, Murata M, Yamauchi K, Harada K. New criteria of normal serum lipid levels in Japanese children: the nationwide study. Pediatr Int. 2002;44: 596–601. 12421254 10.1046/j.1442-200x.2002.01634.x

[11] Urakami T, Morimoto S, Nitadori Y, Harada K, Owada M, Kitagawa T. Urine glucose screening program at schools in Japan to detect children with diabetes and its outcome-incidence and clinical characteristics of childhood type 2 diabetes in Japan. Pediatr Res. 2007;61: 141–145. 17237712 10.1203/pdr.0b013e31802d8a69

[12] Ng M, Fleming T, Robinson M, Thomson B, Graetz N, Margono C, et al Global, regional, and national prevalence of overweight and obesity in children and adults during 1980–2013: a systematic analysis for the Global Burden of Disease Study 2013. Lancet. 2014;384: 766–781. 10.1016/S0140-6736(14)60460-8 24880830 PMC4624264

[13] Cruz ML, Weigensberg MJ, Huang TT, Ball G, Shaibi GQ, Goran MI. The metabolic syndrome in overweight Hispanic youth and the role of insulin sensitivity. J Clin Endocrinol Metab. 2004;89: 108–113. 14715836 10.1210/jc.2003-031188

[14] Yoshinaga M, Tanaka S, Shimago A, Sameshima K, Nishi J, Nomura Y, et al Metabolic syndrome in overweight and obese Japanese children. Obes Res. 2005;13: 1135–1140. 16076981 10.1038/oby.2005.134

[15] Brage S, Wedderkopp N, Ekelund U, Franks PW, Wareham NJ, Andersen LB, et al Features of the metabolic syndrome are associated with objectively measured physical activity and fitness in Danish children: the European Youth Heart Study (EYHS). Diabetes Care. 2004;27: 2141–2148. 15333475 10.2337/diacare.27.9.2141

[16] Ruiz JR, Ortega FB, Rizzo NS, Villa I, Hurtig-Wennlöf A, Oja L, et al High cardiovascular fitness is associated with low metabolic risk score in children: the European Youth Heart Study. Pediatr Res. 2007;61: 350–355. 17314696 10.1203/pdr.0b013e318030d1bd

[17] Ekelund U, Anderssen SA, Froberg K, Sardinha LB, Andersen LB, Brage S, et al Independent associations of physical activity and cardiorespiratory fitness with metabolic risk factors in children: the European youth heart study. Diabetologia. 2007;50: 1832–1840. 17641870 10.1007/s00125-007-0762-5

[18] Dencker M, Thorsson O, Karlsson MK, Lindén C, Wollmer P, Andersen LB. Aerobic fitness related to cardiovascular risk factors in young children. Eur J Pediatr. 2012;171: 705–710. 10.1007/s00431-011-1617-0 22159955

[19] Gulías-González R, Sánchez-López M, Olivas-Bravo A, Solera-Martínez M, Martínez-Vizcaíno V. Physical Fitness in Spanish Schoolchildren Aged 6–12 Years: Reference Values of the Battery EUROFIT and Associated Cardiovascular Risk. J Sch Health. 2014;84: 625–635. 10.1111/josh.12192 25154526

[20] Eisenmann JC, Welk GJ, Ihmels M, Dollman J. Fatness, fitness, and cardiovascular disease risk factors in children and adolescents. Med Sci Sports Exerc. 2007;39: 1251–1256. 17762357 10.1249/MSS.0b013e318064c8b0

[21] Eisenmann JC, Katzmarzyk PT, Perusse L, Tremblay A, Després JP, Bouchard C. Aerobic fitness, body mass index, and CVD risk factors among adolescents: the Québec family study. Int J Obes (Lond). 2005;29: 1077–1083. 15917844 10.1038/sj.ijo.0802995

[22] Eisenmann JC, Welk GJ, Wickel EE, Blair SN. Combined influence of cardiorespiratory fitness and body mass index on cardiovascular disease risk factors among 8–18 year old youth: The Aerobics Center Longitudinal Study. Int J Pediatr Obes. 2007;2: 66–72. 17763013 10.1080/17477160601133713

[23] Suriano K, Curran J, Byrne SM, Jones TW, Davis EA. Fatness, fitness, and increased cardiovascular risk in young children. J Pediatr. 2010;157: 552–558. 10.1016/j.jpeds.2010.04.042 20542285

[24] Adegboye AR, Anderssen SA, Froberg K, Sardinha LB, Heitmann BL, Steene-Johannessen J, et al Recommended aerobic fitness level for metabolic health in children and adolescents: a study of diagnostic accuracy. Br J Sports Med. 2011;45: 722–728. 10.1136/bjsm.2009.068346 20558527

[25] Boddy LM, Thomas NE, Fairclough SJ, Tolfrey K, Brophy S, Rees A, et al ROC generated thresholds for field-assessed aerobic fitness related to body size and cardiometabolic risk in schoolchildren. PLoS One. 2012;7: e45755 10.1371/journal.pone.0045755 23029224 PMC3448715

[26] Olds T, Tomkinson G, Léger L, Cazorla G. Worldwide variation in the performance of children and adolescents: an analysis of 109 studies of the 20-m shuttle run test in 37 countries. J Sports Sci. 2006;24: 1025–1038. 17115514 10.1080/02640410500432193

[27] Léger LA, Mercier D, Gadoury C, Lambert J. The multistage 20 meter shuttle run test for aerobic fitness. J Sports Sci. 1988;6: 93–101. 3184250 10.1080/02640418808729800

[28] Matsuzaka A, Takahashi Y., Yamazoe M., Kumakura N., Ikeda A., Wilk B, et al Validity of the multistage 20-M shuttle-run test for Japanese children, adolescents, and adults. Pediatr Exerc Sci. 2004;16: 113–125.

[29] Jago R, Drews KL, McMurray RG, Thompson D, Volpe SL, Moe EL, et al Fatness, fitness, and cardiometabolic risk factors among sixth-grade youth. Med Sci Sports Exerc. 2010;42: 1502–1510. 10.1249/MSS.0b013e3181d322c4 20139783 PMC2921216

[30] Lee CD, Blair SN, Jackson AS. Cardiorespiratory fitness, body composition, and all-cause and cardiovascular disease mortality in men. Am J Clin Nutr. 1999;69: 373–380. 10075319 10.1093/ajcn/69.3.373

[31] Wei M, Kampert JB, Barlow CE, Nichaman MZ, Gibbons LW, Paffenbarger RS Jr, et al Relationship between low cardiorespiratory fitness and mortality in normal-weight, overweight, and obese men. JAMA. 1999;282: 1547–1553. 10546694 10.1001/jama.282.16.1547

[32] Rizzo NS, Ruiz JR, Hurtig-Wennlöf A, Ortega FB, Sjöström M. Relationship of physical activity, fitness, and fatness with clustered metabolic risk in children and adolescents: the European youth heart study. J Pediatr. 2007;150: 388–394. 17382116 10.1016/j.jpeds.2006.12.039

[33] Hurtig-Wennlöf A, Ruiz JR, Harro M, Sjöström M. Cardiorespiratory fitness relates more strongly than physical activity to cardiovascular disease risk factors in healthy children and adolescents: the European Youth Heart Study. Eur J Cardiovasc Prev Rehabil. 2007;14: 575–581. 17667650 10.1097/HJR.0b013e32808c67e3

